# Daytime bruxism, tardive orofacial dyskinesia, and dysphagia as side effects to duloxetine use over nine years in an octogenarian

**DOI:** 10.1016/j.clinsp.2024.100438

**Published:** 2024-07-22

**Authors:** Carla A. Scorza, Fulvio A. Scorza, Josef Finsterer

**Affiliations:** a Disciplina de Neurociência; Universidade Federal de São Paulo/Escola Paulista de Medicina (UNIFESP/EPM), São Paulo, SP, Brasil; b Neurology Neurophysiology Center, Vienna, Austria

Duloxetine is a commonly prescribed antidepressant from the class of Selective Serotonin-Noradrenaline Reuptake Inhibitors (SSNRI).^1^ It is also approved for the indications of major depression and generalized anxiety disorder. It is usually well tolerated, but, like any medication, occasionally causes side effects.^1^ Common side effects of duloxetine include headache, drowsiness, nausea, dry mouth, decreased appetite, insomnia, anxiety, decreased libido, erectile dysfunction, dizziness, tremor, palpitations, tinnitus, blurred vision, arterial hypertension, digestive problems, abdominal pain, sweating, rash, myalgias, muscle cramps, weight reduction, and urinary retention.^1^ Sudden discontinuation of duloxetine may result in a cataplectic state.^2^ Very rarely, duloxetine has been reported to cause hyperkinetic movement disorders such as tardive dyskinesia,^3^, ^4^, ^5^, ^6^ trismus,^7^ laryngeal dystonia,^8^ akathisia, tremor, restless-leg syndrome,^9^ or opsoclonus-myoclonus syndrome.^10^ Only a few patients with duloxetine-induced bruxism have been reported.^11^, ^12^, ^13^ The combination of tardive dyskinesia, bruxism, and dysphagia has not been reported as an adverse reaction to duloxetine.

The patient is a 83-year-old female, height 162 cm, weight 69 kg who was diagnosed with reactive depression after the death of her husband at the age of 74 and has been on long-term treatment with duloxetine 30 to 60 mg/d since then. Over the years, duloxetine had a positive effect on depressive symptoms, but since the age of 82, she developed orofacial tardive dyskinesias, followed by daytime bruxism, and later by dysphagia. Dysphagia resulted in recurrent aspiration and bronchopulmonary infections. Also notable was a weight loss of 10 kg within the last year prior to presentation. Her medical history was also positive for cataract surgery, hypothyroidism, myocardial infarction, cardiac pacemaker, arterial hypertension, bilateral renal cysts with renal insufficiency, right-sided nephrolithiasis, hyperuricemia, iron deficiency, anemia, hyperlipidemia, and osteoporosis. The family history revealed no evidence of cerebral disease, particularly movement disorders.

Clinical neurologic examination at the age of 83 revealed a fully orientated and cooperative, hypophonic, female with reduced drive, sore neck muscles, hypotelorism, hypoacusis, inversion of the lips, mild bradykinesia, mild discontinuity of the finger-to-nose test, hypoesthesia of the right index finger, and lack of tendon reflexes on the lower limbs. Muscle tone was normal, and she was able to walk unassisted but was unsteady on the Romberg test, the treadmill test, and line walk. She showed a tendency to fall on the blind walk.

Routine blood tests, including toxic, infective, and metabolic studies showed renal insufficiency (creatinine: 1.4 mg/dL (n, 0.5‒1.0 mg/dL), GFR 38 mL/min/1.73 m^2^ (n >90 mL/min/1.73 m^2^), hyperuricemia), iron deficiency, hyperlipidemia, elevated proBNP, slightly elevated liver transaminases, but normal thyroid hormones. Cerebrospinal fluid analysis and electroencephalogram were also normal. Cerebral CT was non-informative and cerebral MRI showed nonspecific gliotic spots.

Therapeutic attempts with biperiden, pregabalin, and amantadine were ineffective. Only after discontinuing duloxetine was a steady improvement in her movement disorders achieved after one month. She also regularly took telmisartan, apixaban, L-thyroxine, depagliflozine, rosuvastatin, and denosumab.

The presented polymorbid octogenarian is interesting for the combination of tardive orofacial dyskinesias, daytime bruxism, and dysphagia due to long-term use of duloxetine over nine years. After discontinuing duloxetine, her extrapyramidal symptoms gradually improved, such that bruxism completely disappeared and the dyskinesias became significantly less. She also noticed improvement in dysphagia and began to gain weight again. Depressive symptoms for which duloxetine had been prescribed nine years previously, did not recur.

Arguments for a causal relationship between duloxetine and the extrapyramidal symptoms are that these decreased or disappeared completely after discontinuation of duloxetine, that bruxism and orofacial dyskinesias were previously reported as a complication of duloxetine, and that the symptoms developed with increasing renal insufficiency, inadequate excretion, drug accumulation, and successive toxic blood levels. Since duloxetine metabolites and duloxetine itself are primarily excreted via the kidney,^14^ it is conceivable that increasing renal failure due to renal cysts led to accumulation of duloxetine metabolites reaching toxic levels.

In summary, this case shows that SSNRIs such as duloxetine can potentially cause drug-induced movement disorders and therefore should be used with caution, especially in the elderly. Such adverse reactions may occur with duloxetine particularly when renal insufficiency leads to accumulation of duloxetine due to decreased elimination of the drug.

## Statement of ethics

a) The study was approved by the institutional review board (responsible: Finsterer J.) at the 4^th^ of November 2022. b) Written informed consent was obtained from the patient for publication of the details of their medical care and any accompanying images.

## Compliance with ethics guidelines

This article is based on previously conducted studies and does not contain any new studies with human participants or animals performed by any of the authors.

## Data availability statement

Data that support the findings of the study are available from the corresponding author.

## Authors’ contributions

JF: Design, literature search, discussion, first draft, critical comments, final approval.

## Funding

No funding was received.

## Conflicts of interest

The author declares that the research was conducted in the absence of any commercial or financial relationships that could be construed as a potential conflict of interest.
